# Lignocellulose-converting enzyme activity profiles correlate with molecular systematics and phylogeny grouping in the incoherent genus *Phlebia* (Polyporales, Basidiomycota)

**DOI:** 10.1186/s12866-015-0538-x

**Published:** 2015-10-19

**Authors:** Jaana Kuuskeri, Miia R. Mäkelä, Jarkko Isotalo, Ilona Oksanen, Taina Lundell

**Affiliations:** Department of Food and Environmental Sciences, Division of Microbiology and Biotechnology, University of Helsinki, Viikki Biocenter 1, P.O.B. 56, FIN-00014 Helsinki, Finland; Department of Forest Sciences, University of Helsinki, Helsinki, Finland

**Keywords:** White rot fungus, Wood decay, Lignocellulose, Lignin biodegradation, Oxidoreductases, Carbohydrate active enzymes, Molecular systematics, Multi-locus phylogeny, *Phlebia*, Polyporales, Basidiomycota

## Abstract

**Background:**

The fungal genus *Phlebia* consists of a number of species that are significant in wood decay. Biotechnological potential of a few species for enzyme production and degradation of lignin and pollutants has been previously studied, when most of the species of this genus are unknown. Therefore, we carried out a wider study on biochemistry and systematics of *Phlebia* species.

**Methods:**

Isolates belonging to the genus *Phlebia* were subjected to four-gene sequence analysis in order to clarify their phylogenetic placement at species level and evolutionary relationships of the genus among phlebioid Polyporales. rRNA-encoding (5.8S, partial LSU) and two protein-encoding gene (*gapdh*, *rpb2*) sequences were adopted for the evolutionary analysis, and ITS sequences (ITS1 + 5.8S + ITS2) were aligned for in-depth species-level phylogeny. The 49 fungal isolates were cultivated on semi-solid milled spruce wood medium for 21 days in order to follow their production of extracellular lignocellulose-converting oxidoreductases and carbohydrate active enzymes.

**Results:**

Four-gene phylogenetic analysis confirmed the polyphyletic nature of the genus *Phlebia*. Ten species-level subgroups were formed, and their lignocellulose-converting enzyme activity profiles coincided with the phylogenetic grouping. The highest enzyme activities for lignin modification (manganese peroxidase activity) were obtained for *Phlebia radiata* group, which supports our previous studies on the enzymology and gene expression of this species on lignocellulosic substrates.

**Conclusions:**

Our study implies that there is a species-level connection of molecular systematics (genotype) to the efficiency in production of both lignocellulose-converting carbohydrate active enzymes and oxidoreductases (enzyme phenotype) on spruce wood. Thus, we may propose a similar phylogrouping approach for prediction of lignocellulose-converting enzyme phenotypes in new fungal species or genetically and biochemically less-studied isolates of the wood-decay Polyporales.

**Electronic supplementary material:**

The online version of this article (doi:10.1186/s12866-015-0538-x) contains supplementary material, which is available to authorized users.

## Background

Fungi of the phylum Basidiomycota have an important role in the global carbon cycle due to their ability to decompose plant biomass that is the richest carbon source on earth. Basidiomycota class Agaricomycetes, in particular the order Polyporales, includes species which are efficient decomposers of wood and other plant biomass, and are able to activate and degrade lignin [1, 2]. The ability to decompose polymeric wood components, that is cellulose, hemicellulose and lignin, requires sets of carbohydrate active enzymes (CAZymes), and oxidoreductases such as peroxidases and laccases [3–5].

The fungal genus *Phlebia* includes several lignin-modifying white rot species which have a high potential for forest-based biotechnology, biopulping, production of lignocellulose-active enzymes and conversion of lignin-derived compounds and xenobiotics [6–15]. Taxonomically, the genus *Phlebia* is positioned to the Polyporales phlebioid clade and to the family Meruliaceae [16–20]. The phlebioid clade includes mainly corticioid basidiocarp-forming species, and the clade consists of seven family names including *Phlebiaceae* originally given by Jülich in 1981 [21]. The genus *Phlebia* has a multitude of species [20, 21] with 203 and 220 taxons recorded in MycoBank (http://www.mycobank.org/) and Index Fungorum (http://www.indexfungorum.org), respectively (August 2015). *Phlebia* has several synonym genera - *Merulius, Mycoaciella* and *Mycoacia* [22, 23].

The type species *Phlebia radiata* Fr. [24] is widely distributed in North America and Europe [25] and has been a subject of genetic and biochemical studies [26–30]. *P. radiata* is a white rot fungus which efficiently degrades lignin in softwood and hardwood [31, 32], depolymerizes milled pine wood [33], mineralizes ^14^C-labelled synthetic lignin (DHP) to carbon dioxide [34, 35], and efficiently produces a versatile set of lignin-modifying oxidoreductases (class II peroxidases and laccase) [26, 28, 30, 35–38]. In addition to *P. radiata*, research has focussed on a few other species of the genus, e.g. *P. tremellosa*, *P. brevispora, P. ochraceofulva* and *P. lindtneri*, in regard to physiology and potential for bioconversion of plant biomass [39–45]. According to genome sequencing of the species *P. brevispora* [2, 4, 21] and *P. radiata* (ongoing) [29], there is a versatile repertoire of genes encoding lignin-modifying and other lignocellulose-converting oxidoreductases, and multiple CAZymes. However, while genomic data may predict the number of genes and potential functions of the extracellular lignocellulose-converting enzymes in fungal species, protein secretion and biochemical enzyme activities need to be verified by proteomics and activity assays, respectively. This is particularly important on natural growth substrates such as wood. Therefore, we performed lignocellulose-converting enzyme activity profiling of 49 *Phlebia* species on wood cultures. The production of lignocellulose-converting enzyme activities were compared with the molecular taxonomy, in order to find out if the enzyme phenotypes of the species groups were determined by their evolutionary proximity and genotype characters.

Our second aim was to deepen the taxonomic knowledge of the phlebioid clade in Polyporales and study the genetic diversity of *Phlebia* by adopting rRNA-encoding (SSU and LSU) and two cellular core protein-encoding genes - glyceraldehyde phosphate dehydrogenase (*gapdh)* and nuclear RNA polymerase II (*rpb2*). The internal transcribed spacer (ITS) sequence has been selected for fungal barcoding and identification [46], giving adequate information for fungal isolate level molecular taxonomy and definition of species. Recently, extensive ITS sequence analysis of phanerochaetoid taxa in the phlebioid clade enlightened the complex phylogeny of this clade [20] and by focusing on the *Phlebia* clade, our study even deepens the understanding of this clade. In our study, statistical and clustering analyses of the *Phlebia* genotype groups with their enzyme activity production profiles demonstrated that the enzyme phenotypes correlated with the species group genotypes. Thus, for the diverse *Phlebia* species, there is a strong connection between the genotype and their CAZyme and lignin-modifying oxidoreductase activity profiles on a natural-like, wood-supplemented growth medium.

## Results

### Molecular identification of *Phlebia* isolates

**Fig. 1:**
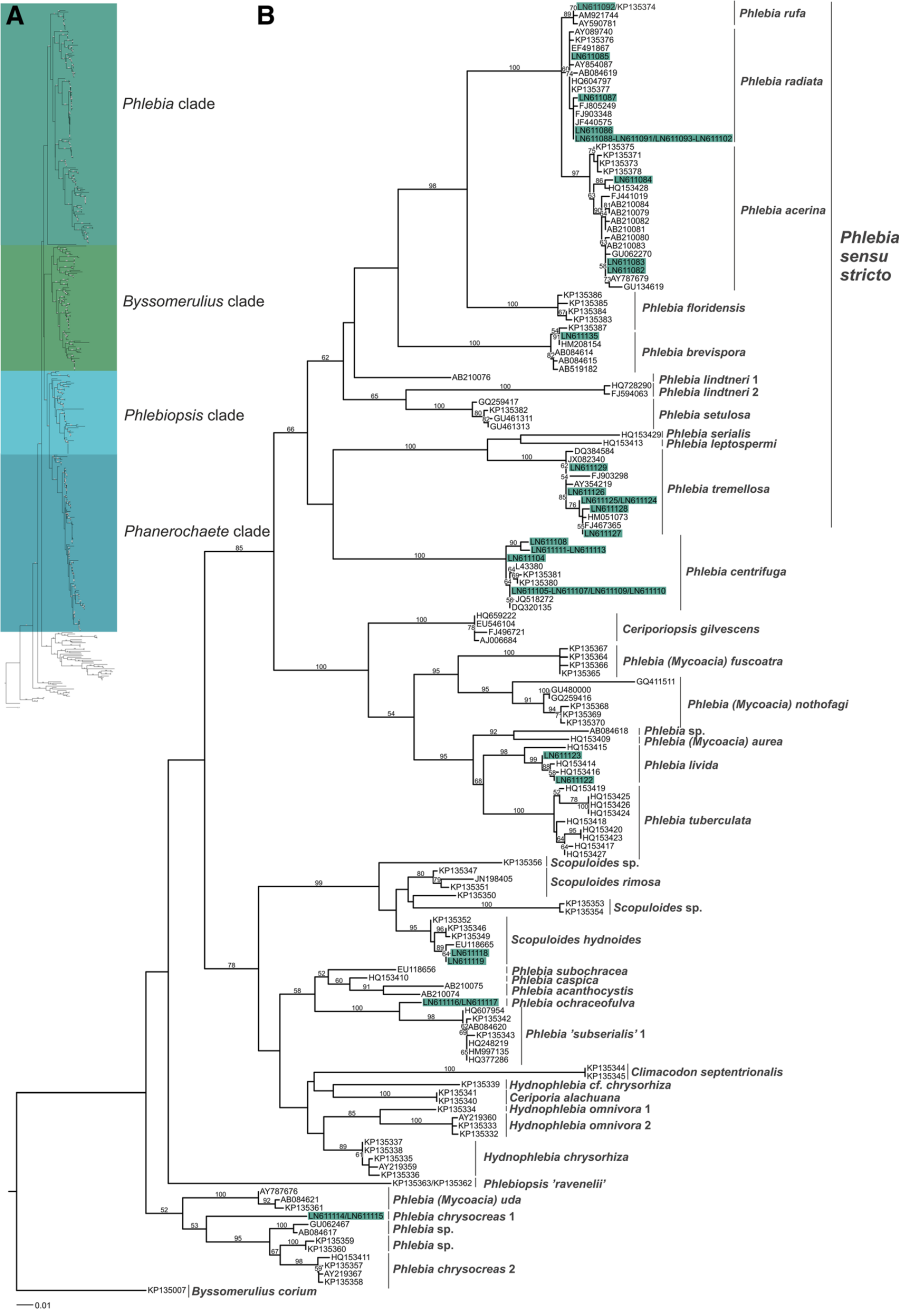
Maximum likelihood trees of the phlebioid clade and *Phlebia* clade of Polyporales based on ITS1-5.8S-ITS2 sequences. (**a**) Maximum likelihood tree illustrating the separation of four clades (*Phlebia, Byssomerulius, Phlebiopsis* and *Phanerochaete*) in the phlebioid clade. For the tree, 481 ITS sequences were aligned and phylogenetical analysis was performed using RAxML v. 7.2.8. and 100x bootstrapping. (**b**) Maximum likelihood analysis of ITS1-5.8S-ITS2 sequences from the *Phlebia* clade. Fungi of this study (shaded in green, ITS accession numbers are presented in Table 1) are compared with related taxons with sequences retrieved from NCBI (http://www.ncbi.nlm.nih.gov/) database. Bootstrap values (100 replications) higher than 50 % are indicated for the nodes. Quotation marks represent uncertain identification or provisional names suggested [20]. An ITS sequence of *Byssomerulius corium* was used as an outgroup. Scale bar represents 0.01 nucleotide substitutions per position

Results obtained from ITS1-5.8S-ITS2 PCR and sequencing of the *Phlebia* isolates confirmed their earlier identification results, which were mostly based on their basidiocarp morphological features, with a few exceptions (Additional file 1: Table S1). Most of the FBCC (University of Helsinki Fungal Biotechnology Culture Collection) isolates previously identified to the species *P. radiata* were correctly confirmed including 14 isolates which were 100 % identical according to their complete ITS sequences (Fig. 1). The only exceptions were the isolates FBCC4 and FBCC345, which were over 99 % identical to the species *P. acerina* (Additional file 1: Table S1). In addition, the phylogenetic maximum likelihood analysis strongly supported positioning of the two isolates in the *P. acerina* branch (bootstrap value 97, Fig. 1) and thereby, these isolates were re-named *P. acerina* at the species level in this study.

Also, the isolates FBCC421 and FBCC426 were re-named *P. centrifuga* and *P. subserialis*, respectively, according to their ITS-sequence identity (99.0 % and 99.8 %) in comparison to taxon reference sequences (Additional file 1: Table S1) and support from high node bootstrap values (100 and 100) (Fig. 1 and Additional file 2: Figure S1a). Considering *P. subserialis,* our isolate FBCC426 and one reference sequence were positioned far away from *Phlebia* species into the *Phanerochaete* clade. Our ITS-sequencing and phylogenetic analyses were unable to confirm the previous identification for three isolates of the 54 studied. Isolate FBCC427 (initially *P. subserialis*) was positioned in the *Phlebiopsis* clade but distant from *Phlebiopsis*, *Rhizochaete* and *Phaeophlebiopsis* (Additional file 2: Figure S1b). Isolate FBCC296 (initially *P. albida*) was distantly related to the *Phlebia* clade and was situated in the *Phanerochaete* clade. However, more information is apparently needed to confirm the species level taxonomy, and therefore, these isolates were not yet given definite identities or taxon names, and are thus depicted *Phlebia* sp. isolates (Additional file 1: Table S1).

### ITS phylogeny

An ITS sequence dataset was generated for phylogenetic analyses of the Polyporales phlebioid clade by including reference sequences retrieved from NCBI GenBank and the sequences of this study. Altogether 481 ITS sequences were included in the maximum likelihood (ML) phylogram (Fig. 1a), and 156 sequences were positioned in the *Phlebia* clade (Fig. 1b). The phylogenetic analyses resulted in three major clades in the phlebioid clade, which were named according to Floudas and Hibbet [20] as *Phlebia*, *Byssomerulius* and *Phanerochaete* clades. Similarly as in the recent study [20], the *Phanerochaete* clade was divided into *Phlebiopsis* and *Phanerochaete* clades.

According to the ITS phylogeny, genus *Phlebia* produced no single taxonomic cluster (Fig. 1). While *Phlebia* species are widely distributed in the ITS tree, the *Phlebia sensu stricto* species form one uniform core group, which includes the type species *P. radiata* (Fig. 1b). The three species *P. radiata*, *P. acerina* and *P. rufa* are very closely related forming a distinct branch (bootstrap value 100) in the *Phlebia* clade. In addition, *Phlebia sensu stricto* includes the species *P. floridensis, P. brevispora, P. lindtneri, P. setulosa, P. serialis, P. leptospermi* and *P. tremellosa*. It is noteworthy that the *Phlebia* clade includes a number of isolates that were identified to the genera *Ceriporiopsis, Scopuloides*, *Climacodon, Phlebiopsis, Ceriporia* and *Hydnophlebia* (Fig. 1b)*.*

Furthermore, the species *P. unica, P. firma,* and two isolates of *P. subserialis* were clearly separated from the *Phlebia* clade and were positioned in *Phanerochaete* or *Phlebiopsis* clades (Additional file 2: Figure S1). Three isolates without a previous species-level identity and thus named *Phlebia* sp. were similarly positioned outside the *Phlebia* clade.

### Four-gene phylogeny

**Fig. 2:**
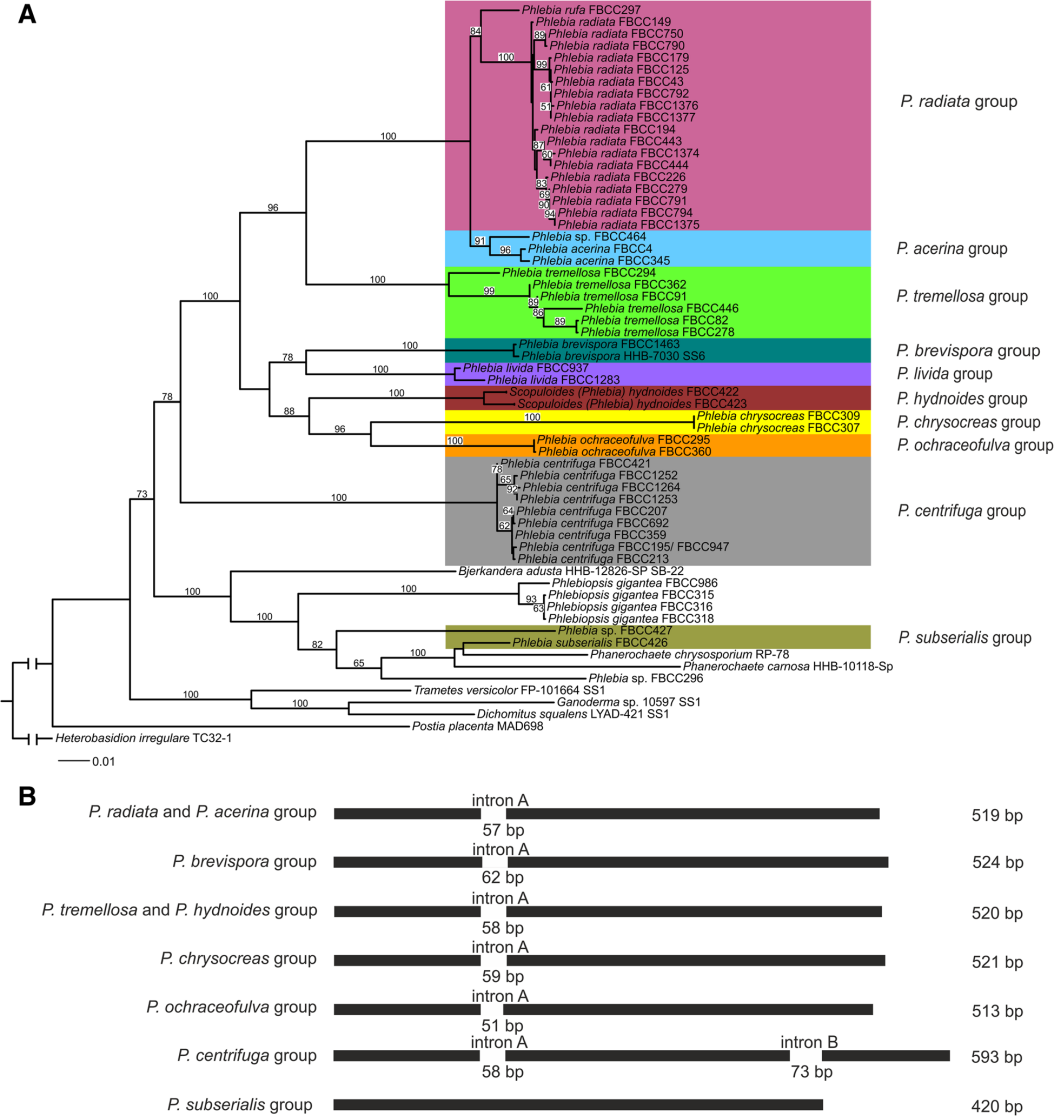
Maximum likelihood phylogeny and exon-intron structure of partial *gapdh* nucleotide sequences of the *Phlebia* isolates. (**a**) Maximum likelihood phylogeny of the *Phlebia* isolates showing the phylogroups formed. 5.8S, partial LSU, and partial sequences from two protein-encoding genes (*gapdh*, *rpb2*) were concatenated for an alignment, and the phylogenetic analysis was performed using RAxML v. 7.2.8. and 100x bootstrapping. Sequences of related Agaricomycetes species (taxons without FBCC-identifier) were retrieved from JGI MycoCosm database [76] and NCBI (http://www.ncbi.nlm.nih.gov/). Species names are followed by isolate culture collection identifiers. The sequences from species *Heterobasidion irregulare* (Russulales, Basidiomycota) were used as an outgroup. Bootstrap values higher than 50 % are indicated for the nodes. Scale bar represents 0.01 nucleotide substitutions per position. (**b**) Exon-intron structure of partial *gapdh* nucleotide sequences from the *Phlebia* phylogroups studied. Black and white areas indicate exons and introns, respectively

According to the four-gene multilocus phylogeny analysis, *Phlebia* isolates were divided into ten phylogroups (Fig. 2a, Table 1). Statistical analyses of the enzyme activity data were based on this grouping except for *P. brevispora* due to only one isolate cultivated for enzyme profiling. The first phylogroup included isolates of the species *P. radiata* and *P. rufa* (Fig. 2a). The well-supported sister lineage to this phylogroup was the *P. acerina* branch consisting of three isolates. According to the four-gene phylogeny, *P. tremellosa* clearly deviated from the *P. radiata* and *P. acerina* species groups with 100 % branching support (Fig. 2a). The species *P. brevispora* and *P. livida,* as well as *P. hydnoides, P. chrysocreas* and *P. ochraceofulva* all branched as sister lineages forming distinct species clusters or clades, and were therefore treated as separate phylogroups in the statistical enzyme-phenotype analyses.

**Table 1:** Fungal isolates of this study

Group name^a^	Fungal Biotechnological Culture Collection identifier	Identity^b^	Site of origin	Natural substrate^c^	ITS Accession number	Isolate number used in Figs. 4 and 5
*P. radiata* group	43	*Phlebia radiata*	Finland; Vantaa	D	LN611085	1
	297	*Phlebia rufa*	Sweden		LN611092	2
	125	*Phlebia radiata*	Finland; Lieksa	D	LN611086	3
	149	*Phlebia radiata*	Finland; Ruovesi	D	LN611087	4
	179	*Phlebia radiata*	Finland; Lammi	D	LN611088	5
	194	*Phlebia radiata*	Finland; Sodankylä	D	LN611089	6
	226	*Phlebia radiata*	Finland; Kolari	D	LN611090	7
	279	*Phlebia radiata*	Sweden		LN611091	8
	443	*Phlebia radiata*	UK	D	LN611093	9
	444	*Phlebia radiata*	France		LN611094	10
	750	*Phlebia radiata*	Finland; Lammi		LN611095	11
	790	*Phlebia radiata*	Finland; Ruovesi	D	LN611096	12
	791	*Phlebia radiata*	Finland; Ruovesi	D	LN611097	13
	792	*Phlebia radiata*	Finland; Ruovesi	D	LN611098	14
	794	*Phlebia radiata*	Finland; Ruovesi	D	LN611099	15
	1374	*Phlebia radiata*	Finland; Lammi	D	LN611100	16
	1375	*Phlebia radiata*	Finland; Ruovesi	D	LN611101	17
	1376	*Phlebia radiata*	unknown		LN611102	18
	1377	*Phlebia radiata*	unknown		LN611103	19
*P. acerina* group	4	*Phlebia acerina*	unknown		LN611082	20
	345	*Phlebia acerina*	Russia		LN611083	21
	464	*Phlebia* sp.	Argentina; Bariloche	D	LN611084	22
*P. brevispora* group	1463	*Phlebia brevispora*	USA; Florida		LN611135	23
*P. tremellosa* group	82	*Phlebia tremellosa*	Finland; Salo	D	LN611124	24
	91	*Phlebia tremellosa*	Finland; Perniö	D	LN611125	25
	294	*Phlebia tremellosa*	Canada	D	LN611127	26
	362	*Phlebia tremellosa*	Russia; Kavalerovo	D	LN611128	27
	446	*Phlebia tremellosa*	Netherlands		LN611129	28
	278	*Phlebia tremellosa*	Sweden		LN611126	29
*P. livida* group	937	*Phlebia livida*	Finland; Lammi	C	LN611122	30
	1283	*Phlebia livida*	Norway; Telemark	C	LN611123	31
*P. hydnoides* group	423	*Phlebia (Scopuloides) hydnoides*	Belgium; Bois de Matignolle	D	LN611119	32
	422	*Phlebia (Scopuloides) hydnoides*	France; Haute Savoie		LN611118	33
*P. chrysocreas* group	307	*Phlebia chrysocreas*	unknown		LN611114	34
	309	*Phlebia chrysocreas*	unknown		LN611115	35
*P. ochraceofulva* group	295	*Phlebia ochraceofulva*	Sweden		LN611116	36
	360	*Phlebia ochraceofulva*	Sweden		LN611117	37
*P. centrifuga* group	207	*Phlebia centrifuga*	Finland; Kolari	C	LN611105	38
	213	*Phlebia centrifuga*	Finland; Aakenus	C	LN611106	39
	195	*Phlebia centrifuga*	Finland; Sodankylä	C	LN611104	40
	359	*Phlebia centrifuga*	Sweden		LN611107	41
	692	*Phlebia centrifuga*	Finland; Sodankylä	C	LN611109	42
	947	*Phlebia centrifuga*	Finland; Kolari	C	LN611110	43
	1252	*Phlebia centrifuga*	Bulgaria; Rila mountains		LN611111	44
	1253	*Phlebia centrifuga*	Bulgaria; Rila mountains		LN611112	45
	1264	*Phlebia centrifuga*	Bulgaria; Rila mountains		LN611113	46
	421	*Phlebia centrifuga*	USA; Idaho	C	LN611108	47
*P. subserialis* group	426	*Phlebia subserialis*	France		LN611120	48
	427	*Phlebia* sp.	France; Rhône		LN611121	49
Species included in phylogenetic study	296	*Phlebia* sp.	Sweden		LN611130	
	315	*Phlebiopsis gigantea*	Sweden		LN611131	
	316	*Phlebiopsis gigantea*	Sweden		LN611132	
	318	*Phlebiopsis gigantea*	Sweden		LN611133	
	986	*Phlebiopsis gigantea*	Finland; Kolari	C	LN611134	

^a^Confirmed by ITS1-5.8S-ITS2 and LSU sequence similarity using nBLAST search. See details in Methods

^b^The isolates were grouped based on ITS sequence similarity and phylogrouping based on phylogenetic analyses of concatenated SSU, partial LSU sequences, and partial sequences from two protein-encoding genes (*gapdh, rpb2*)

^c^C = Coniferous wood, D = Deciduous wood

Isolates of *P. radiata*, *P. tremellosa*, *P. centrifuga* and *P. subserialis* also diverged at the species level (Fig. 2a). However, the *P. subserialis* group was formed by only two isolates, and more noteworthy, the isolate FBCC426 is the nearest related to species of *Phanerochaete* (*P. chrysosporium* and *P. carnosa*, bootstrap value 100 %). Moreover, the two *Phanerochaete* species, *Phlebia subserialis*, and the isolates *Phlebia* sp. FBCC296 and FBCC427 were positioned far out from the *Phlebia sensu stricto*, and in fact, these isolates were the most related to the species *Phlebiopsis gigantea* and *Bjerkandera adusta* (Fig. 2a).

Presence or absence of introns, intron positioning and intron length varied in *Phlebia gapdh* genes with respect to the species grouping (Fig. 2b). The *P. radiata* and *P. acerina* phylogroups had similar *gapdh* exon-intron structures and length of the sequenced region. *P. tremellosa* and *P. hydnoides* phylogroups were similarly uniform. Other phylogroups showed variable sizes of *gapdh* PCR products due to differences in intron length and positioning. All *P. centrifuga gapdh* sequences had a unique intron B, whereas isolate FBCC427 from the *P. subserialis* group as well as *Phlebia* sp. FBCC296 and all *Phlebiopsis gigantea* isolates lacked both introns A and B. With the *gapdh* primers used, no PCR-product was obtained for the *P. livida* isolates, which leaves the question open whether this species group has a more variable *gapdh* gene structure than the other studied species. In general, exon-intron structure of the *gapdh* gene (Fig. 2b) was coherent with the multilocus sequence phylogeny and phylogrouping of *Phlebia* species.

Phylogenetic analyses conducted with either individual or contiguous ITS and partial LSU sequences, and respectively with individual or concatenated *gapdh* and *rpb2* sequences, resulted in evolutionary trees with slightly different topologies than was obtained with the four-gene phylogeny (Additional file 3: Figure S2, Additional file 4: Figure S3). Phylogenetic analyses based on ITS and *gapdh* sequences positioned *P. brevispora* near to *P. radiata - P.acerina* sister species, when the LSU and *rpb2* sequences were not able to confirm its evolutionary placement (Additional file 4: Figure S3). Our four-gene phylogeny also positioned *P. brevispora* closer to *P. livida* than to *P. radiata*. Positioning of *P. livida* as well as *P. hydnoides* was not supported by the protein-encoding sequences (Additional file 3: Figure S2b). Taken together, similar fungal species-based phylogroupings were observed in all evolutionary analyses.

### Fungal growth rates and activity normalization

In order to test if the enzyme activities were influenced by the differences in fungal growth rates, we tried to estimate production of mycelium biomass (as mycelium dry weight) for each isolate and each culture flask in the end of cultivation. However, deviation of the dry weight values between the parallel cultures (three parallel culture flasks) was too divergent. This was probably due to wood sawdust particles that were attached to the mycelia. Instead, we measured the hyphal growth rate on malt agar plates for each isolate, and used these values (cm d^−1^) (Additional file 5: Figure S4f) to adjust the enzyme activity values (μkat l^−1^) of day 14. This normalization resulted in fairly similar differences between the isolates and species groups that was observed with the non-normalized enzyme activities, except for a few isolates of *P. centrifuga* (see below).

### Production of enzyme activities

**Fig. 3:**
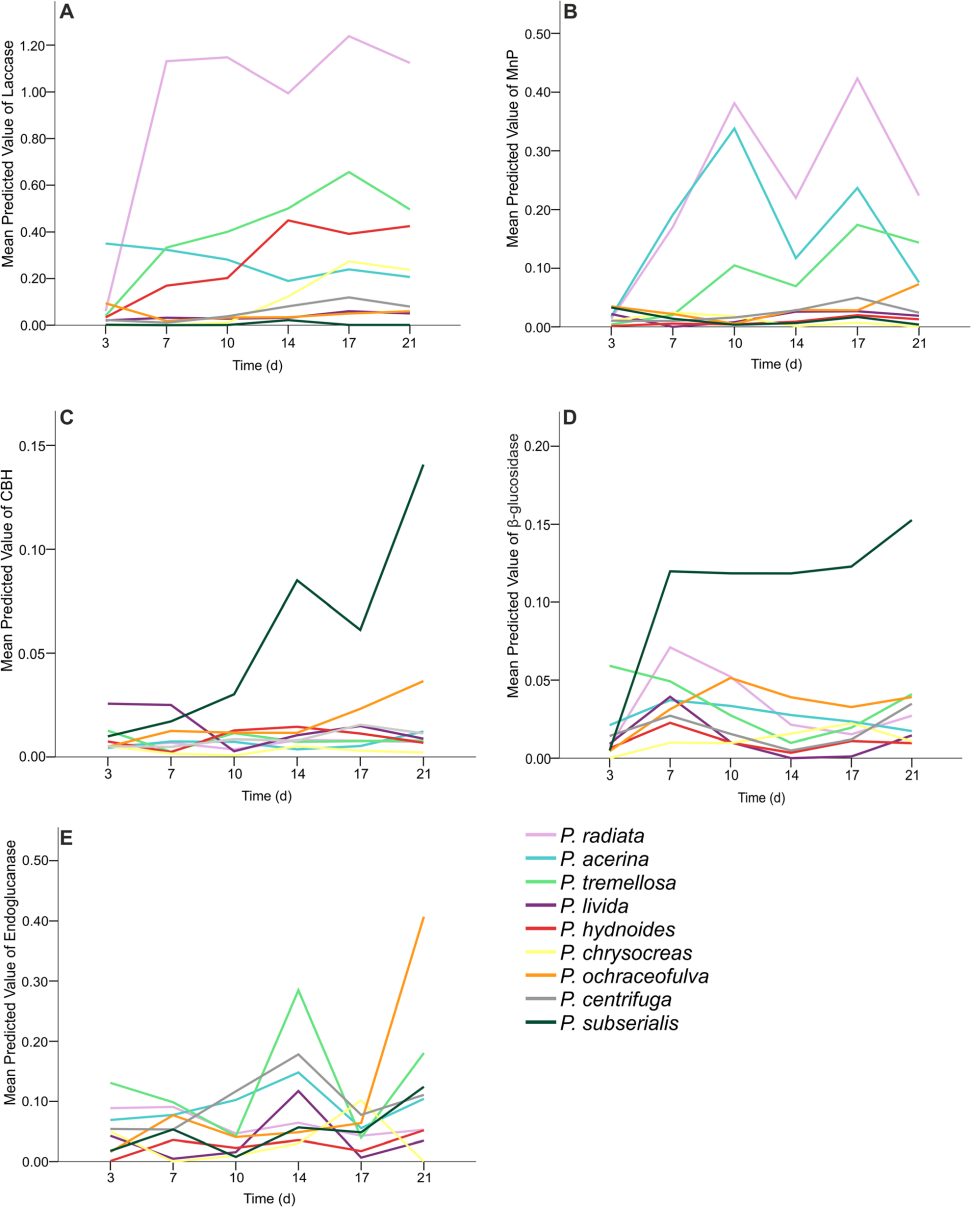
Fitted values of enzyme activities of each phylogenetic group. Fitted values (mean predicted value) of (**a**) laccase (**b**) MnP (**c**) CBH (**d**) β-glucosidase and (**e**) endoglucanase activities of each phylogenetic group during 21 days of cultivation in semi-solid milled spruce cultures

**Fig. 4:**
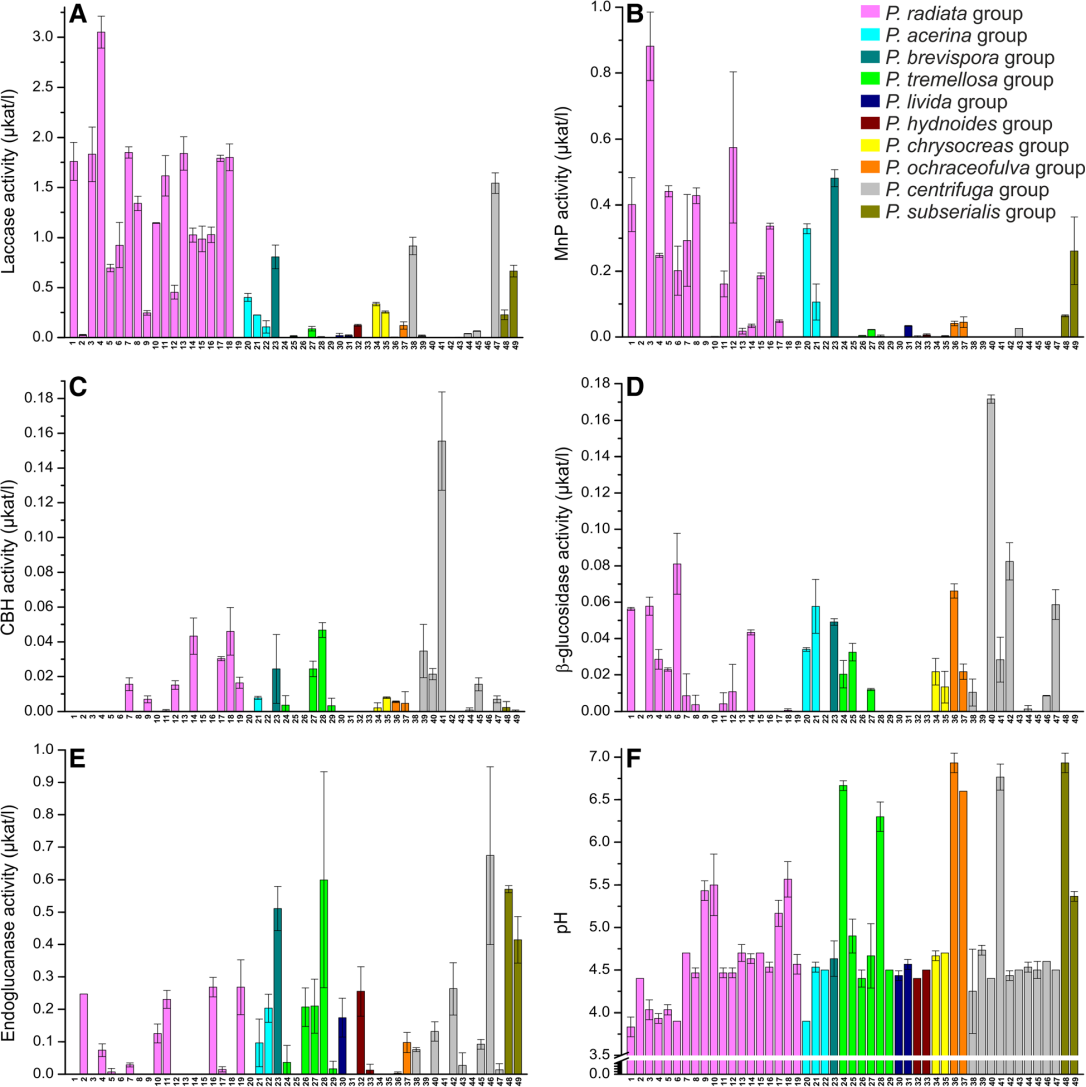
Enzyme activities and pH values of culture liquids of *Phlebia* isolates. Extracellular (**a**) laccase (**b**) MnP (**c**) CBH (**d**) β-glucosidase and (**e**) endoglucanase activities after 14 days of cultivation in semi-solid milled spruce cultures of *Phlebia* isolates*.* (**f**) pH values of the semi-solid milled spruce culture liquids after 21 days of cultivation. Initial pH of the culture medium was 4.5. Error bars represent standard deviation of the mean activity value or pH value from two parallel cultivations. The isolates were numbered as listed in Table 1

During the 21 days of cultivation on semi-solid liquid medium with milled spruce as a carbon source, all the 49 *Phlebia* isolates produced lignocellulose-converting enzyme activities periodically (Fig. 3). When the enzyme activity patterns were investigated on the 14^th^ day of cultivation, differences between *Phlebia* phylogroups became apparent (Fig. 4). The *P. radiata* group produced the highest levels of oxidoreductase activities, that is laccase and manganese peroxidase (MnP) (up to 3.0 and 0.9 μkat l^-1^, respectively) (Fig. 4a, b). The highest laccase activity, 3.0 μkat l^-1^, was observed in the cultures of *P. radiata* FBCC149, whereas *P. radiata* FBCC125 produced the highest MnP activity (0.9 μkat l^-1^). Relatively high laccase and MnP activites were detected in the cultures of *P. brevispora* FBCC1463. Even though the overall production of laccase in the *P. centrifuga* phylogroup was moderate, one isolate (FBCC421) in this group attained similar activity levels (maximum 1.5 μkat l^-1^) as obtained in the *P. radiata* – *P. acerina* phylogroups. However, with normalized laccase activities another isolate of *P. centrifuga* (FBCC207) demonstrated the highest production value on the day 14, which is due to its very slow hyphal growth rate (Additional file 5: Figures S4a, S4f). In the case of MnP activity, normalization of the data (on day 14) caused minor differences, with an exceptionally high value for one slow-growing isolate of *P. centrifuga* (FBCC947) (Additional file 5: Figures S4b, S4f).

In contrast to the lignin-modifying oxidoreductases, the activity production profiles of the hydrolytic CAZymes were more coherent within each phylogroup (Fig. 3), and less evident differences were detected in the CAZyme activity levels between the fungal isolates of each phylogroup (Fig. 4). Concerning cellulose-degrading enzyme activities, the highest level of endoglucanase activity was detected after two weeks for the isolates *P. tremellosa, P. centrifuga* and *P. subserialis* (Fig. 4e), peaking up to 0.7 μkat l^-1^ in the culture liquid of *P. centrifuga* FBCC1264. Cellobiohydrolase (CBH) activities in turn were marginal, and the highest values (0.16 μkat l^-1^) were observed for the *P. centrifuga* phylogroup (Fig. 4c), which was furthermore obvious with the normalized activity values (Additional file 5: Figure S4c). The highest β-glucosidase activity (0.17 μkat l^-1^) was also produced in the *P. centrifuga* phylogroup (Fig. 4d). Activities of β-glucosidase in *P. radiata, P. acerina*, *P. brevispora, P. tremellosa* and *P. ochraceofulva* phylogroups were at similar levels but isolate-level differences within each of the phylogroups were detected (Fig. 4d). When CBH activities were studied, the *P. radiata* species group shared similar production patterns as *P. acerina, P. tremellosa* and *P. hydnoides* groups (Fig. 4c), and endoglucanase activities (Fig. 4e) were at the same levels in *P. radiata, P. tremellosa* and *P. subserialis* phylogroup cultures. Isolate-level differences among the species groups were also observed in hyphal growth rates on ME agar (Additional file 5: Figure S4f).

This study utilized generalized estimating equations (GEE) method to analyze differences resulting from enzyme activity values of the samples taken and measured at sequential time points. When the complete cultivation period (21 d) was studied, statistically significant differences in production of lignocellulose-converting oxidoreductases and cellulolytic enzyme activities were detected between the phylogroups (Additional file 6: Table S2). In the statistical calculations, time and species group were the explanatory variables, and also their interaction was statistically significant. When fitted values of enzyme activities of each phylogroup were plotted, the high variation of laccase activity production levels between the phylogroups was observed (Fig. 3). *P. radiata* group produced the highest activities of laccase and MnP during the cultivation period. The second best producer of laccase activity were the *P. tremellosa* and *P. hydnoides* phylogroups which produced increasing amounts of laccase activity within the course of the cultivation. Together with the *P. radiata* phylogroup, the *P. acerina* and *P. tremellosa* groups produced higher amounts of MnP activity compared to the other phylogroups. Fitted values of enzyme activities of each phylogroup showed moderate production of cellulolytic activities. The phylogenetically most distant and incoherent group, the *P. subserialis* group, produced the highest CBH and β-glucosidase activities when compared to the other *Phlebia* phylogroups.

### pH values and culture acidity

The pH values of the culture fluids remained stable during the 21 d cultivation period for most of the fungal isolates (Fig. 4f). However, a few of the *P. radiata* isolates (FBCC43, FBCC149, and FBCC194) and *P. acerina* isolate FBCC4 apparently acidified their cultures leading to final pH values below 4.0, which suggests active production of organic acids. On the contrary, final pH values in the cultures of *P. tremellosa* isolates FBCC446 and FBCC82, *P. ochraceofulva* isolates FBCC360 and FBCC295*, P. centrifuga* isolate FBCC359, and *P. subserialis* isolate FBCC426 increased to pH values over 6 (pH 6.3-6.9).

### Enzyme phenotype clusters

**Fig. 5:**
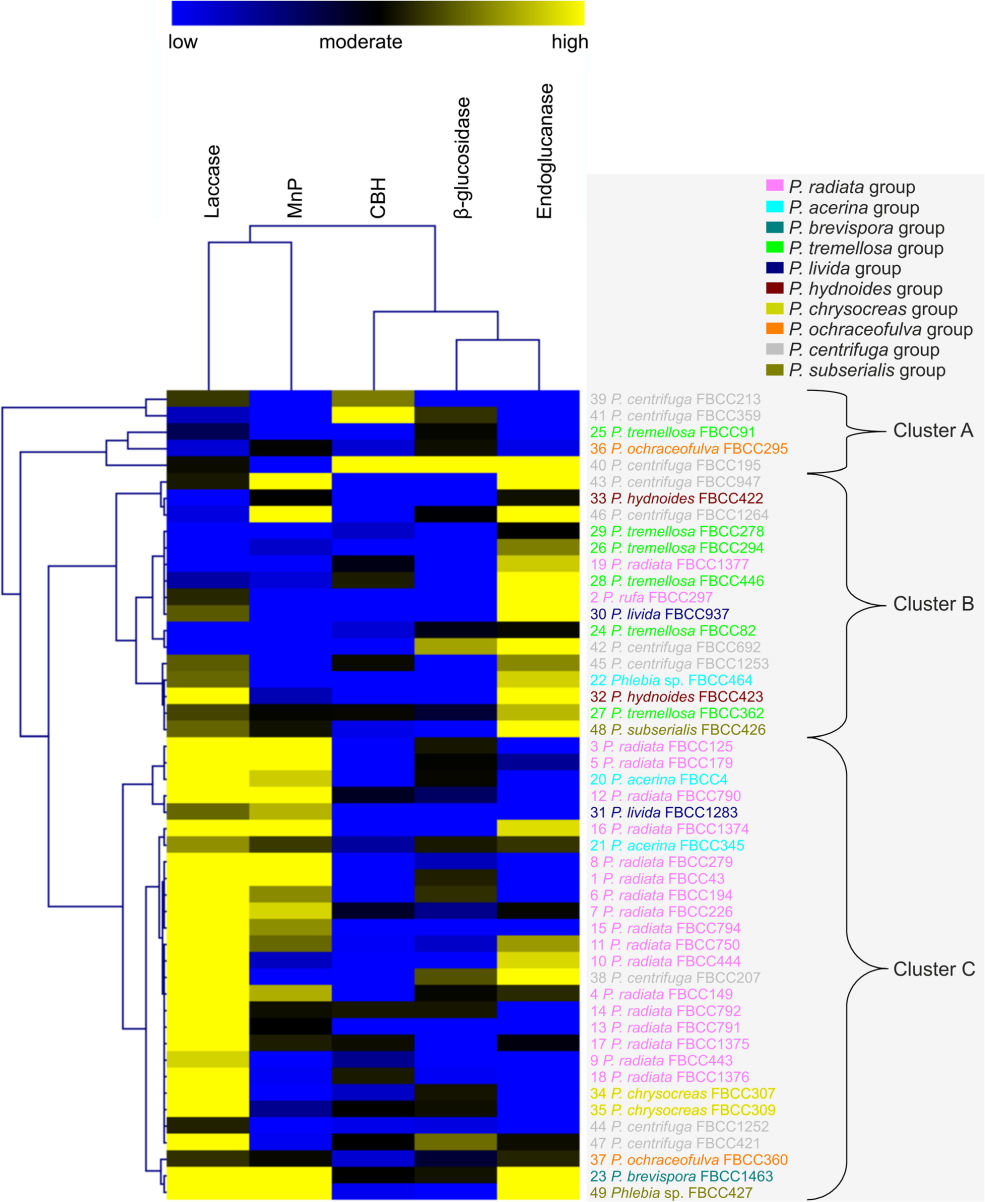
Hierarchical clustering of the *Phlebia* isolates. Hierarchical clustering presentation of lignocellulose-converting enzyme activities from fungal cultures on milled spruce wood on day 14. The normalized values taking into account the hyphal growth rates were used for calculations. The isolates were numbered as listed in Table 1

To further visualize and compare the plant-biomass degrading enzyme production profiles as combinations of the periodical enzyme activity values of the fungal isolates, a double hierarchical clustering calculation method was adopted. Similarities of enzyme activities in the semi-solid milled spruce cultures for each sampling day were calculated to create the data matrix. The normalized enzyme activity values on cultivation day 14 were selected for presentation (Fig. 5).

According to the normalized enzyme activity profiles at this time point, isolates of *Phlebia* demonstrated three enzyme phenotype clusters (Fig. 5). Cluster C contained most of the isolates, including isolates of *P. radiata* and *P. acerina*, and this cluster demonstrated production of both laccase and MnP activities. Cluster B showed high endoglucanase activities and contained sixteen isolates. In Cluster A, enzyme activity production was more scattered but included the highest production of cellulose-degrading CBH activities. Overall, clustering analysis pinpointed two enzyme production patterns: *Phlebia* isolates producing high oxidoreductase (laccase and MnP) activities, and isolates showing high activities of cellulose-degrading enzymes (CBH, endoglucanase, β-glucosidase).

## Discussion

In this study, we report on the interdependence of fungal molecular systematics (genotyping) and extracellular enzyme activity profiles (enzyme phenotyping) for isolates of ten species of the largely unknown genus *Phlebia* and other representatives of the phlebioid clade of Polyporales. The 49 fungal isolates were subjected to multilocus gene phylogeny, and cultivated on semi-solid spruce wood medium to follow wood-decay enzyme activities for a three-week period.

Besides enzyme production profiling, our second attempt was to examine molecular systematics of the taxonomically incoherent genus *Phlebia*, and to more accurately position the type species (*P. radiata*) in the genus and phlebioid clade. The genus *Phlebia* has been proposed to be a set of unrelated taxa that have some shared morphological traits [47]. Our sequence-based phylogenetic study was also conducted in order to confirm taxonomic species-level identity of phlebioid and *Phlebia* isolates with previous history of principally morphology-based identification.

Several studies – both traditional and modern molecular systematics applying - have tried to resolve the taxonomy of the multiple genera positioned in the phlebioid clade of Polyporales, but so far without complete success [21, 22, 48–52]. The recent study on phanerochaetoid fungi increased this knowledge but showed the need for reference sequences for some of the species. Our study provided 152 new sequences, and the phylogenetic analyses, both multilocus alignment and single-gene phylogenetic analysis, produced phylograms which point out that fungi with taxon species name *Phlebia* are found in most of the currently recognized lineages of the phlebioid clade (order Polyporales, class Agaricomycetes) [21].

In our study, the barcode marker sequence [46] demonstrated its usefulness for concluding phylogenetic positioning of evolutionarily closely and more distantly related species of *Phlebia*. Although the ITS region is useful to resolve fungal phylogenetic relationships to certain extent, the importance of using other non-protein and protein-encoding genes to resolve the phylogenetic position of certain *Phlebia* species has been demonstrated [47, 49, 50]. For these reasons, we included three genes - rRNA LSU, and protein-encoding *gapdh* and *rpb2* - to improve the outcome of our molecular systematic and evolutionary analyses.

Species named as *Phlebia* can be found in other clades of Polyporales, for example the species *P. bresadolae* and *P. queletii* belong to the ‘residual polyporoid clade’ [21]. It has been described earlier that the *Phlebia* clade is not uniformly composed of only *Phlebia* species [20]. This study confirmed that the *Phlebia* clade includes also fungal isolates identified to the genera *Ceriporiopsis, Scopuloides*, *Climacodon, Phlebiopsis, Ceriporia* and *Hydnophlebia.* This demonstrates the difficulty to obtain a uniform phylogenetic analysis on *Phlebia* species. For that reason, extensive ITS phylogeny was used as a starting point for generating *Phlebia*, *Phanerochaete* and *Phlebiopsis* clades, wherein our isolates were positioned. After analyzing the *Phlebia* clade, our study confirmed the existence of the *Phlebia sensu stricto* [20]. According to our ITS analysis we propose that at least *P. lindtneri*, *P. serialis* and *P. leptospermi* should be added to this core group. It remains unclear, if *P. centrifuga* belongs to the core group since other phylogenetic analyses of this study and other studies on *P. centrifuga* [20, 49] are not supporting this positioning.

Species-level identity of most of our fungal isolates was confirmed by the four-gene and ITS sequence phylogeny analyses, and taxonomic re-positioning occurred only for a few *Phlebia*-named isolates. Two isolates (FBCC4, FBCC345) previously identified as *P. radiata* were re-classified to *P. acerina* due to their high ITS sequence identity (99.4-99.5 %) to *P. acerina* isolates. Sampling of the reference ITS sequences of *P. radiata*, *P. acerina* and *P. rufa* taxons obtained from NCBI showed that some of these isolates were incorrectly named. Difficulty to identify and discriminate these three species by using traditional methods is not a surprise since *P. rufa*, *P. acerina* and *P. radiata* are very similar in their basidiocarp (basidiomal) and hymenial macro-structure and micro-morphology [25], thus also supporting their genetic similarity and evolutionary close speciation.

*P. chrysocreas* isolates of this study (FBCC307, FBCC309) were separated from the four reference *P. chrysocreas* isolates according to ITS sequence phylogeny*.* Four reference sequence isolates without species-level identity (named as *Phlebia* sp.) fall in between this rather scattered branch. *P. ochraceofulva* isolates (FBCC 295 and FBCC 360) produced a separated lineage without reference sequences. Their identity is problematic to confirm without more reference taxons.

Another peculiarity is the positioning of the isolate *P. subserialis* FBCC426 in our phylogenetic analyses, which supported clustering of the isolate far from the *Phlebia* clade to the *Phanerochaete* clade. Different taxonomic positioning of isolates of *P. subserialis* has been observed in earlier studies [21, 47, 49–51, 53]. According to our ITS phylogeny, there is a *Phlebia subserialis* lineage (number 1) in the *Phlebia* clade and a second lineage in the *Phanerochaete* clade (number 2). Recently, a third *P. subserialis* lineage has been demonstrated in the *Phlebia* clade [20]. Six *P. subserialis* ITS sequences were positioned in the *Phanerochaete* clade, but they were separated into two lineages (Additional file 2: Figures S1a, S1b). The first lineage includes our isolate FBCC426. A provisional species name of *Phanerochaete krikophora* was given to the second lineage [20].

We cultivated the phlebioid isolates on semi-solid medium containing milled Norway spruce wood, which is a natural lignocellulose substrate for a multitude of Polyporales wood-decay species in the northern temperate and boreal forests. Most of the *Phlebia* species prefer angiosperm wood for growth but may also colonize dead gymnosperm wood [25, 48]. For instance *P. centrifuga* is usually observed as a saprotroph of Norway spruce [54]. So far, production and activities of wood-decay enzymes has been reported only for a few species of the phlebioid clade. In our study, the wood-containing medium supported production of lignin-modifying oxidoreductase and CAZyme activities in species of *Phlebia*.

In general, moderate levels of cellulolytic endoglucanase activity were produced by all phlebioid isolates, and the highest activities were measured after two weeks of growth. Production of low endoglucanase activities on wood cultures by *P. radiata* and *P. tremellosa* isolates was demonstrated earlier [55], and negligible amounts of other cellulolytic activities have been observed for *P. radiata* cultures on lignocellulose substrates [26]. The type species *P. radiata* produces several cellulolytic enzymes, including β-1,4-endoglucanase, exo-β-1,4-glucanase, aryl-β-1,4-glucosidase, and β-1,4-glucosidase [56], hemicellulolytic enzymes, including β-xylosidase and endo-1,4-β-xylanase [57], and debranching enzymes, such as α-glucuronidase and α-galactosidase, which may cleave the glucosyl side-chains of hemicelluloses and pectin [58, 59]. In this respect, it was expected that production of a wide array of CAZymes acting on wood polysaccharides would be as general as in *P. radiata* at least among the *Phlebia sensu stricto* species. The measured CAZyme activities were reasonably coherent within the species phylogroups, and the few observed differences between fungal isolates (intraspecies variation) may be a consequence of differences in the hyphal growth rates of the isolates.

According to enzyme activity production profiling, *P. subserialis* isolate (FBCC426) and most of the isolates of *P. acerina* and *P. radiata* clustered differently in the double hierarchical clustering calculation analysis. Also statistical analyses showed that the *P. subserialis* phylogroup produced higher cellulolytic enzyme (CBH and β-glucosidase) activities during the cultivation period compared to species that were included in the *Phlebia sensu stricto*. Phenotype similarity of *P. subserialis* to the genus *Phanerochaete* is well supported in this context, since *Phanerochaete* species (*P. chrysosporium*, *P. carnosa*, *P. sordida*) are well known producers of cellulolytic enzymes, with several CAZymes and respective genes characterized [60–62].

Considering the lignin-modifying oxidoreductases, our study reveals that there are significant differences in production of laccase activities among the *Phlebia* species groups. Production of laccase activity was one of the features clearly distinguishing between the enzyme phenotype groups. This is rather surprising since production of laccase has classically categorised wood-decay fungi as white rot and lignin-modifying species [63]. However, in line with the accumulating genomic data and comparative genomics on Basidiomycota and Polyporales species, the role of laccase in decomposition of wood lignin has been questioned [3, 64]. Instead, it is more evident that secreted class II heme peroxidases and in particular, various MnPs are necessary for lignin degradation and white rot type of wood decay [1, 2].

In this respect, it was assumed that all phlebioid isolates studied could actively produce MnP when growing on spruce wood. Convincingly, MnP activities were either at moderate steady levels throughout the cultivation period, or a pattern of cyclic production (MnP activity peaking on 10th and 17th cultivation day) was observed for closely related *P. radiata*, *P. acerina* and *P. tremellosa* strains. Cyclic production of MnP has been reported for *P. radiata* isolate FBCC43 on milled alder wood under similar cultivation conditions [28]. Furthermore, high MnP activities as well as protein properties for MnP enzymes (long- and short-MnPs) and isoenzymes have been reported for several *Phlebia* species (*P. radiata, P. tremellosa, P. brevispora, P. floridensis, P. subserialis*, *Phlebia* sp. MG60, and *Phlebia* sp. b19) [8, 42, 43, 65–67], and divergent *mnp* genes have been cloned from e.g. *P. radiata* [27].

Surprisingly, no lignin peroxidase (LiP) activity was detected in the spruce wood cultures of any of the phlebioid isolates studied, although isolates of *P. radiata, P. tremellosa, P. floridensis, P. brevispora* and *P. ochraceofulva* produced LiP enzymes under variant culture conditions and in cultures including solid lignocellulose supplements [8, 28, 41, 42, 67]. For *P. radiata*, LiP activity has been reported even on similar semi-solid cultures but supplemented with alder sawdust [28, 30], and three LiP-encoding genes have been cloned and characterized in this species [37]. Partial *lip* gene sequences were amplified from isolates of *P. tremellosa* and *P. chrysocreas* [68]. In several previous studies [38, 66, 69] the authors have discussed that LiP activities may not be detectable due to the presence of coloured, apparently phenolic compounds, which are dissolved in the fungal cultures from the wood and plant biomass substrates. These type of compounds may have masked LiP activities also in our study.

Our ITS sequence phylogeny analysis was in agreement with the recent extensive ITS phylogeny study on taxa of *Phanerochaete* and related genera [20]. The protein-encoding gene (*gapdh* and *rpb2*) regions, however, were somewhat less successful in supporting evolutionary positioning of our set of *Phlebia* isolates. The *gapdh* primers designed and applied in this study resulted in a higher frequency of PCR amplification than obtained with *rpb2* primers. Accordingly, *gapdh* intron positioning was one of the genotyping features most conserved among the *Phlebia sensu stricto* species. Presence of a unique second intron in *gapdh* genes of *P. centrifuga* isolates differentiated this species from *Phlebia sensu stricto*. One challenge in using the *gapdh* region for molecular systematics and phylogenetic analyses is yet the lack of reference sequences in nucleotide sequence databases. For this reason, current use of primers targeted to ITS sequences and rRNA encoding genes together with carefully selected conserved protein-encoding genes promotes coherency for taxonomic comparison and fungal systematics.

## Conclusions

Our study on the polyphyletic genus *Phlebia* infers that the fungal phylogroups showed significant differences in lignocellulose-converting enzyme phenotypes according to generalized estimation statistical analysis. These results may reflect different efficiencies of the enzyme-production profiles of *Phlebia* species in their natural habitats, and predict their life-style differences on strategies to degrade various types of wood and lignocellulose. Knowledge of the taxonomy and physiological versatility of genus *Phlebia* has a great importance for more applicative studies on fungal enzyme production and bioconversion abilities. Our study is the first using such approach of combined molecular genotyping and enzyme activity profiling, and may thus be an example for similar research for systematically unknown or biochemically less studied wood-decay fungi, and aid in characterizing new fungal species and isolates.

## Methods

### Fungal isolates

The fungal isolates (Table 1) were living pure cultures deposited in the University of Helsinki Fungal Biotechnology Culture Collection (FBCC, fbcc@helsinki.fi), of the Division of Microbiology and Biotechnology, Department of Food and Environmental Sciences.

### Cultivation of the fungal isolates

Fungal isolates (Table 1) were maintained on 2 % (w/v) malt-extract (Biokar Diagnostics, France) agar (2 % w/v agar-agar, Biokar Diagnostics, France) (MEA) plates at room temperature. For extraction of DNA, fungal isolates were cultivated on 2 % MEA plates for 14 days at 28 °C. For the determination of hyphal growth rates, one mycelium agar plug (7 mm in diameter) was inoculated in the center of each 2 % MEA plate and cultivated for 14 days at 28 °C - except in the case of the fungal isolates FBCC297, FBCC464, FBCC1283, FBCC422, FBCC423, FBCC359 and FBCC421, which were cultivated at 22 °C. For enzyme activity production, *Phlebia* spp. strains were cultivated as semi-solid liquid cultures in three parallel flasks containing 100 ml of low-nitrogen asparagine-succinate medium, pH 4.5 [31, 35], without glucose but supplemented with 1 g (dry weight) of milled Norway spruce (*Picea abies*) wood as the sole carbon source. The semi-solid cultures were inoculated with four mycelial agar plugs (7 mm in diameter) from 7–14 days grown MEA plates, and incubated for 21 days at 28 °C in the dark as stationary cultures.

### DNA extraction

Pieces of mycelia were disrupted with acid-washed and sterilized glass beads (1–2 mm) in sterile plastic cryotubes using FastPrep®-24 Instrument (M.P. Biomedicals, USA). DNA was extracted by using CTAB buffer and purified as previously described [27]. Amount and quality of total DNA was determined with NanoDrop 1000 Spectrophotometer (Thermo Scientific, Germany).

### PCR amplification

Complete nuclear rDNA ITS region (ITS1 + 5.8S + ITS2), part (1361–1419 bp) of the large rRNA subunit (LSU) coding region, partial (505–636 bp) sequence of the glyceraldehyde phosphate dehydrogenase encoding gene (*gapdh*), and a ca. 1097 bp region of the 140 kDa size subunit of the nuclear RNA polymerase II encoding gene (*rpb2*) were PCR amplified by using genomic DNA as template. The complete ITS region was amplified with ITS1 and ITS4 primers [70], the 5′ region of the LSU with 5.8sr and LR7 primers [71], and the partial *rpb2* region with 7cf and 11bR primers [72]. Primers were designed to amplify the partial *gapdh* region from *Phlebia* isolates (fw: 5′-ATG GTC TAC ATG TTC AAG TAC GAC-3′; rev: 5′-TCG ACG AGG GGA TGA TGT T -3′). PCR reactions were conducted with Dynazyme II or Phusion Hot Start DNA polymerase (Finnzymes, Finland). PCR was performed as previously described [27, 73].

### Sequencing

The amplified PCR products were either directly used as templates or cut out of the agarose gels and purified with GeneJET™ Gel Extraction Kit (Fermentas, Lithuania), and used for sequencing (Institute of Biotechnology, University of Helsinki, Finland, and Macrogen Ltd, Republic of Korea) with the initial PCR primer pairs.

### Sequence analyses

Nucleotide sequences were edited and assembled with BioEdit software [74]. Regions of ITS1, 5.8S and ITS2 were identified with the ITS extractor software [75]. Introns were excluded manually from the protein-encoding *gapdh* sequences in all analyses. They were confirmed by recognizing the consensus exon/intron splice junction sequences present in reference genes. Reference sequences were obtained from NCBI GenBank (http://www.ncbi.nlm.nih.gov), especially the ITS sequences produced by Floudas and Hibbet [20], and JGI MycoCosm genome portal (http://genome.jgi.doe.gov/programs/fungi/index.jsf, [76], Additional file 7: Table S3). All sequences were aligned using PRANK (http://www.ebi.ac.uk/goldman-srv/webprank/) with the default settings [77]. The alignments were manually trimmed (overhangs were removed and gaps were corrected) prior to phylogenetic analyses.

After multiple alignment of each trimmed gene, ITS alignment comprising the regions ITS1, 5.8S and ITS2 of 481 DNA sequences from taxa of the phlebioid clade was created and subjected to maximum likelihood (ML) inference by using RAxML v. 7.2.8 (http://phylobench.vital-it.ch/raxml-bb/, [78]). The best-scoring ML tree was searched and the bootstrap analysis was run under the GTRCAT model, using 100 rapid bootstrap replicates. Trees were visualized with the Interactive Tree Of Life (iTOL) online tool [79] and CorelDRAW X3 software (Corel Corporation, Canada). The resulted ML tree helped to divide ITS sequences into four subsets. The ITS sequences of each subset were realigned separately using PRANK and the ML analyses were performed with the same parameters in each case. Multilocus phylogenetic analysis based on 5.8S (SSU) (158 nucleotides), and LSU (1421 nucleotides), *gapdh* (413 nucleotides) and *rpb2* (913 nucleotides) gene coding regions were conducted from the aligned dataset of 62 combined nucleotide sequences containing 2905 positions, of which 810 were variable (including missing data). ITS1 and ITS2 sequences were omitted from the four-gene phylogeny since these were poorly aligned. ML analysis was performed for this alignment with RAxML with GTRCAT model of evolution. Node support was assessed with 100 rapid bootstrap replicates. Individual runs were also performed for each target sequence and for combined ribosomal (ITS + LSU) sequences and combined protein-encoding sequences (*gapdh* + *rpb2*). The ML analyses were performed with the same parameters in each case.

### Determination of enzyme activities

Enzyme activities from samples collected on days 3, 7, 10, 14, 17, 21 and 28 after inoculation from three semi-solid culture flasks were measured by using 96-well plates and Tecan Infinite M200 microplate reader spectrophotometer (Tecan, USA) for each fungal isolate. Reaction volume was 250 μl, and three parallel reactions were measured for each sample and each fungal culture flask.

Laccase activity was determined by following the oxidation of 1 mM 2,6-dimethoxyphenol (2,6-DMP, Aldrich, Germany) at 476 nm in 50 mM Na-malonate buffer (pH 4.5) at 25 °C [28, 80]. MnP activity was assayed by detecting the formation of Mn^3+^-malonate complex at 270 nm in 50 mM Na-malonate buffer (pH 4.5) at 25 °C [81].

Cellulase (cellobiohydrolase I, β-glucosidase and endo-β-1,4-glucanase) reactions were performed in 50 mM Na-citrate buffer (pH 5) at 45 °C [82]. Cellobiohydrolase (CBHI) activity was measured by using 4-methylumbelliferyl-β-D-lactoside (MULac, Biokemis, Russia) as substrate. β-glucosidase activity was assayed by quantification of *p-*nitrophenol released from 1 mM 4-nitrophenyl β-D-glucopyranoside (Applied Chemical Laboratories, USA) at 400 nm. Endo-β-1,4-glucanase activity was determined with 1 % (wt/vol) hydroxyethyl cellulose (HEC, Sigma, USA) as a substrate. Reducing sugars were measured with dinitrosalisylic acid (DNS) at 540 nm [82].

For calculation of the hyphal growth rate, mean data points (measured from three parallel MEA plates) were selected from the linear growth phase. This was presented as cm d^−1^. Enzyme activity values on cultivation day 14 were divided by this value to obtain the ‘normalized’ enzyme activity values.

### Statistical analyses

The linear models and the method of generalized estimating equations (GEE) were used to analyze differences in the set of enzyme activities between the *Phlebia* phylogroups. The phylogroups were determined by the multigene sequence similarity and evolutionary analysis. In each generalized linear model, time and group were explanatory variables and their interaction terms were also included in all models. The enzyme activities were assumed to follow the Tweedie distribution with link function chosen to be the log link. The working correlation matrix of within-subject repeated measurements was assumed to have a first-order autoregressive structure in each model. In estimation, the index parameter of the Tweedie distribution was first estimated by using the R software 3.1.1 (R Core Team, 2014) with the tweedie package. Then the GEE procedure was performed by using IBM SPSS Statistics 22, release 22.0.0.0 (IBM Corporation, USA). Significance level of 5 % was used in all analyses.

To visualize normalized enzyme activity profiles of the 49 *Phlebia* isolates after 14 days of growth on semi-solid milled spruce medium, hierarchical clustering of the enzyme activities was performed by generating a Pearson correlation matrix with Multiexperiment Viewer (MeV) [83].

### Availability of supporting data

The data sets supporting the results of this article are included within the article and its additional files. All nucleotide sequences were deposited in EMBL-EBI European Nucleotide Archive (ENA) under accession numbers presented in Additional file 1: Table S1 [EMBL: LN610995-LN611135 and LN651202-LN651212].

## Additional files

Additional file 1: Table S1. Morphological and sequence based identification of isolates and sequenced specimens used in this study. (PDF 152 kb) Additional file 2: Figure S1. Maximum likelihood analysis of ITS sequences of (a) *Phanerochaete* and (b) *Phlebiopsis* lineages of the phlebioid clade. Description: Bootstrap values (100 replications) higher than 50 % are indicated for the nodes. Fungi of this study (shaded in blue) are compared with related taxons with sequences retrieved from NCBI (http://www.ncbi.nlm.nih.gov/) database. Quotation marks represent uncertain identification or provisional names [20]. Scale bar represents 0.01 nucleotide substitutions per position. (TIFF 4093 kb) Additional file 3: Figure S2. Maximum likelihood tree of *Phlebia* isolates and related species. Description: Partial nucleotide sequences from (a) rRNA-encoding genes (ITS1-5.8S-ITS2, LSU) and (b) two protein-encoding genes (*gapdh*, *rpb2*) were concatenated for alignment, and the phylogenetic analysis was performed using RAxML v. 7.2.8. and 100x bootstrapping for the nodes. For comparison, sequences from JGI MycoCosm database [76] and NCBI were retrieved. Species names are followed by isolate culture collection identifiers or sequence accessions. Bootstrap values higher than 50 % are indicated for the nodes. Scale bar represents 0.01 nucleotide substitutions per position. (TIFF 3310 kb) Additional file 4: Figure S3. Phylogenetic trees of phlebioid isolates from maximum likelihood analyses of individual gene datasets. Description: Bootstrap values (100 replications) higher than 50 % are indicated for the nodes. Species names are followed by culture collection identifiers. For comparison, sequences from JGI MycoCosm database [76] were retrieved. Scale bar represents 0.01 nucleotide substitutions per position. (TIFF 6079 kb) Additional file 5: Figure S4. Normalized enzyme activities of culture liquids and hyphal growth rates of *Phlebia* isolates. Description: Normalized extracellular (a) laccase (b) MnP (c) CBH (d) β-glucosidase and (e) endoglucanase activities on day 14 in semi-solid milled spruce cultures of *Phlebia* isolates*.* Error bars represent standard deviation of the mean activity value from two parallel cultivations. (f) Hyphal growth rates from three parallel MEA plates. Mean value for each isolate is presented. Error bars represent variance of the growth rates. The isolates were numbered as listed in Table 1. (TIFF 1427 kb) Additional file 6: Table S2. Statistical tests of model effects and estimates of index parameter in Tweedie distribution. (PDF 155 kb) Additional file 7: Table S3. Accessions for nucleotide and protein-encoding gene model sequences used for comparison in the four-gene phylogenetic analyses. Description: All the sequences were retrieved from JGI MycoCosm database [76] with minor exception: ^a^ from NCBI http://www.ncbi.nlm.nih.gov/. (PDF 13 kb)

## Abbreviations

CAZymes: carbohydrate active enzymes
CBH: cellobiohydrolase
FBCC: University of Helsinki Fungal Biotechnology Culture Collection
*gapdh*: gene encoding for glyceraldehyde phosphate dehydrogenase
GEE: generalized estimating equations
ITS: internal transcribed spacer
LiP: lignin peroxidase
LSU: large rRNA subunit
MEA: malt extract agar
ML: maximum likelihood
MnP: manganese peroxidase
*rpb2*: gene encoding for a nuclear RNA polymerase II subunit
SSU: small rRNA subunit
