# Supplementary material for: Lignocellulose-converting enzyme activity profiles correlate with molecular systematics and phylogeny grouping in the incoherent genus Phlebia (Polyporales, Basidiomycota)
Source: BMC Microbiol. 2015 Oct 19;15:217. doi: 10.1186/s12866-015-0538-x (PMC4610053; doi:10.1186/s12866-015-0538-x)
Supplement: Additional file 1: Table S1. — Morphological and sequence based identification of isolates and sequenced specimens used in this study. (PDF 152 kb) [file 12866_2015_538_MOESM1_ESM.pdf]

Table S1. Morphological and sequence based identification of isolates and sequenced specimens used in this study.

FBCC number	Morphological identification	Sequence based identification	Molecular identification	Highest identities of nBLAST[b]	Max identity (%) (BLAST)	New name of the isolate	ITS+ LSU	*gapdh*	*rpb2*
4	*Phlebia radiata*	*Phlebia acerina*	ITS[a]	*Phlebia acerina* (AB210080)	99.4	*Phlebia acerina*	LN611 082	LN611 042	LN610 995
345	*Phlebia radiata*	*Phlebia acerina*	ITS	*Phlebia acerina* (AB210080)	99.5	*Phlebia acerina*	LN611 083	LN611 043	LN610 996
464	*Phlebia* sp.	*Phlebia sp.*	ITS	*Phlebia rufa* (HQ153428)	99		LN611 084	LN611 044	LN610 997
43	*Phlebia radiata*	*Phlebia radiata*	ITS	*Phlebia radiata* (AY854087)	99.8		LN611 085	LN611 045	LN610 998
125	*Phlebia radiata*	*Phlebia radiata*	ITS	*Phlebia radiata* (AY854087)	99.5		LN611 086	LN611 046	LN610 999
149	*Phlebia radiata*	*Phlebia radiata*	ITS	*Phlebia radiata* (AY854087)	99.5		LN611 087	LN611 047	LN611 000
179	*Phlebia radiata*	*Phlebia radiata*	ITS	*Phlebia radiata* (AY854087)	99.7		LN611 088	LN611 048	LN611 001
194	*Phlebia radiata*	*Phlebia radiata*	ITS	*Phlebia radiata* (AY854087)	99.7		LN611 089	LN611 049	LN611 002
226	*Phlebia radiata*	*Phlebia radiata*	ITS	*Phlebia radiata* (AY854087)	99.7		LN611 090	LN611 050	LN611 003
279	*Phlebia radiata*	*Phlebia radiata*	ITS	*Phlebia radiata* (AY854087)	99.7		LN611 091	LN611 051	LN611 004
297	*Phlebia rufa*		ITS	*Phlebia rufa* (KP135374)	100		LN611 092	LN611 052	LN611 005
443	*Phlebia radiata*	*Phlebia radiata*	ITS	*Phlebia radiata* (AY854087)	99.7		LN611 093	LN611 053	LN611 006
444	*Phlebia radiata*	*Phlebia radiata*	ITS	*Phlebia radiata* (AY854087)	99.7		LN611 094	LN611 054	LN611 007
750	*Phlebia radiata*	*Phlebia radiata*	ITS	*Phlebia radiata* (AY854087)	99.7		LN611 095	LN611 055	LN611 008
790	*Phlebia radiata*	*Phlebia radiata*	ITS	*Phlebia radiata* (AY854087)	99.7		LN611 096	LN611 056	LN611 009
791	*Phlebia radiata*	*Phlebia radiata*	ITS	*Phlebia radiata* (AY854087)	99.7		LN611 097	LN611 057	LN611 010
792	*Phlebia radiata*	*Phlebia radiata*	ITS	*Phlebia radiata* (AY854087)	99.7		LN611 098	LN611 058	LN611 011
794	*Phlebia radiata*	*Phlebia radiata*	ITS	*Phlebia radiata* (AY854087)	99.7		LN611 099	LN651 204	

Table S1. Continued.

FBCC number	Morphological identification	Sequence based identification	Molecular identification	Highest identities of nBLAST[b]	Max identity (%) (BLAST)	New name of the isolate	ITS+ LSU	*gapdh*	*rpb2*
1374	*Phlebia radiata*	*Phlebia radiata*	ITS	*Phlebia radiata* (AY854087)	99.4		LN611 100	LN611 059	LN611 012
1375	*Phlebia radiata*	*Phlebia radiata*	ITS	*Phlebia radiata* (AY854087)	99.7		LN611 101	LN611 060	LN611 013
1376	*Phlebia radiata*	*Phlebia radiata*	ITS	*Phlebia radiata* (AY854087)	99.7		LN611 102	LN611 061	LN611 014
1377	*Phlebia radiata*	*Phlebia radiata*	ITS	*Phlebia radiata* (AY854087)	99.7		LN611 103	LN611 062	LN611 015
195	*Phlebia centrifuga*	*Phlebia centrifuga*	ITS	*Phlebia centrifuga* (L43380)	99.5		LN611 104	LN651 206	LN611 016
207	*Phlebia centrifuga*	*Phlebia centrifuga*	ITS	*Phlebia centrifuga* (L43380)	99.5		LN611 105	LN651 205	LN611 017
213	*Phlebia centrifuga*	*Phlebia centrifuga*	ITS	*Phlebia centrifuga* (L43380)	99.5		LN611 106	LN611 063	LN611 018
359	*Phlebia centrifuga*	*Phlebia centrifuga*	ITS	*Phlebia centrifuga* (L43380)	99.7		LN611 107	LN651 207	LN611 019
421	*Phlebia albida*	*Phlebia centrifuga*	ITS	*Phlebia centrifuga* (JQ358815)	99	*Phlebia centrifuga*	LN611 108	LN611 064	
692	*Phlebia centrifuga*	*Phlebia centrifuga*	ITS	*Phlebia centrifuga* (L43380)	99.5		LN611 109	LN651 208	LN611 020
947	*Phlebia centrifuga*	*Phlebia centrifuga*	ITS	*Phlebia centrifuga* (L43380)	99.5		LN611 110	LN651 209	LN611 021
1252	*Phlebia centrifuga*	*Phlebia centrifuga*	ITS	*Phlebia centrifuga* (JQ358815)	99		LN611 111	LN651 211	LN611 022
1253	*Phlebia centrifuga*	*Phlebia centrifuga*	ITS	*Phlebia centrifuga* (JQ358815)	99		LN611 112	LN651 212	LN611 023
1264	*Phlebia centrifuga*	*Phlebia centrifuga*	ITS	*Phlebia centrifuga* (JQ358815)	99		LN611 113	LN651 210	LN611 024
307	*Phlebia chrysocreas*	*Phlebia chrysocreas*	ITS	*Phlebia chrysocreas* (HQ153411)	92		LN611 114	LN611 065	LN611 025
309	*Phlebia chrysocreas*	*Phlebia chrysocreas*	ITS	*Phlebia chrysocreas* (HQ153411)	92		LN611 115	LN611 066	LN611 026
295	*Phlebia ochraceofulva*		LSU	*Phlebia subochracea* (EU118656)	96		LN611 116	LN651 202	LN611 027

Table S1. Continued

FBCC number	Morphological identification	Sequence based identification	Molecular identification	Highest identities of nBLAST[b]	Max identity (%) (BLAST)	New name of the isolate	ITS+ LSU	*gapdh*	*rpb2*
360	*Phlebia ochraceofulva*		LSU	*Phlebia subochracea* (EU118656)	96		LN611117	LN651203	LN611028
422	*Phlebia hydnoides*	*Scopuloides hydnoides*	ITS	*Scopuloides hydnoides* (EU118665)	99	*Scopuloides (Phlebia) hydnoides*	LN611118	LN611067	LN611029
423	*Phlebia hydnoides*	*Scopuloides hydnoides*	ITS	*Scopuloides hydnoides* (EU118665)	98.4	*Scopuloides (Phlebia) hydnoides*	LN611119	LN611068	LN611030
426	*Phlebia ochraceofulva*	*Phlebia subserialis*	ITS	*Phlebia subserialis* (FJ791134)	99.8	*Phlebia subserialis*	LN611120		
427	*Phlebia subserialis*		LSU	*Rhizochaete filamentosa* (AY219393)	97	*Phlebia* sp.	LN611121	LN611069	LN611031
937	*Phlebia livida*	*Phlebia livida*	ITS	*Phlebia livida* (HQ153414)	99.3		LN611122		LN611032
1283	*Phlebia livida*	*Phlebia livida*	ITS	*Phlebia livida* (HQ153414)	99.5		LN611123		LN611033
82	*Phlebia tremellosa*	*Phlebia tremellosa*	ITS	*Phlebia tremellosa* (HM051073)	99		LN611124	LN611070	LN611034
91	*Phlebia tremellosa*	*Phlebia tremellosa*	ITS	*Phlebia tremellosa* (HM051073)	99		LN611125	LN611071	
278	*Phlebia tremellosa*	*Phlebia tremellosa*	ITS	*Phlebia tremellosa* (HM051073)	98		LN611126	LN611072	LN611035
294	*Phlebia tremellosa*	*Phlebia tremellosa*	ITS	*Phlebia tremellosa* (HM051073)	99		LN611127	LN611073	
362	*Phlebia tremellosa*	*Phlebia tremellosa*	ITS	*Phlebia tremellosa* (HM051073)	99		LN611128	LN611074	
446	*Phlebia tremellosa*	*Phlebia tremellosa*	ITS	*Phlebia tremellosa* (DQ384584)	99		LN611129	LN611075	LN611036
Isolates included in the phylogenetic study									
296	*Phlebia albida*		ITS	*Phanerochaete sordida* (FJ481018)	93	*Phlebia* sp.	LN611130	LN611076	LN611037
315	*Phlebiopsis gigantea*	*Phlebiopsis gigantea*	ITS	*Phlebiopsis gigantea* (AF87485)	99.4		LN611131	LN611077	LN611038
316	*Phlebiopsis gigantea*	*Phlebiopsis gigantea*	ITS	*Phlebiopsis gigantea* (AF87485)	99.4		LN611132	LN611078	LN611039

Table S1. Continued

FBCC number	Morphological identification	Sequence based identification	Molecular identification	Highest identities of nBLAST[b]	Max identity (%) (BLAST)	New name of the isolate	ITS+ LSU	*gapdh*	*rpb2*
318	*Phlebiopsis gigantea*	*Phlebiopsis gigantea*	ITS	*Phlebiopsis gigantea* (AF87486)	99.4		LN611 133	LN611 079	LN611 040
986	*Phlebiopsis gigantea*	*Phlebiopsis gigantea*	ITS	*Phlebiopsis gigantea* (AF087487)	99.8		LN611 134	LN611 080	
1463	*Phlebia brevispora*	*Phlebia brevispora*	ITS	*Phlebia brevispora* (HM208154)	100		LN611 135	LN611 081	LN611 041

[a]ITS1-5.8s-ITS2 gene region

[b]accession numbers in brackets
