# Supplementary figures and images for: Lignocellulose-converting enzyme activity profiles correlate with molecular systematics and phylogeny grouping in the incoherent genus Phlebia (Polyporales, Basidiomycota)
Source: BMC Microbiol. 2015 Oct 19;15:217. doi: 10.1186/s12866-015-0538-x (PMC4610053; doi:10.1186/s12866-015-0538-x)

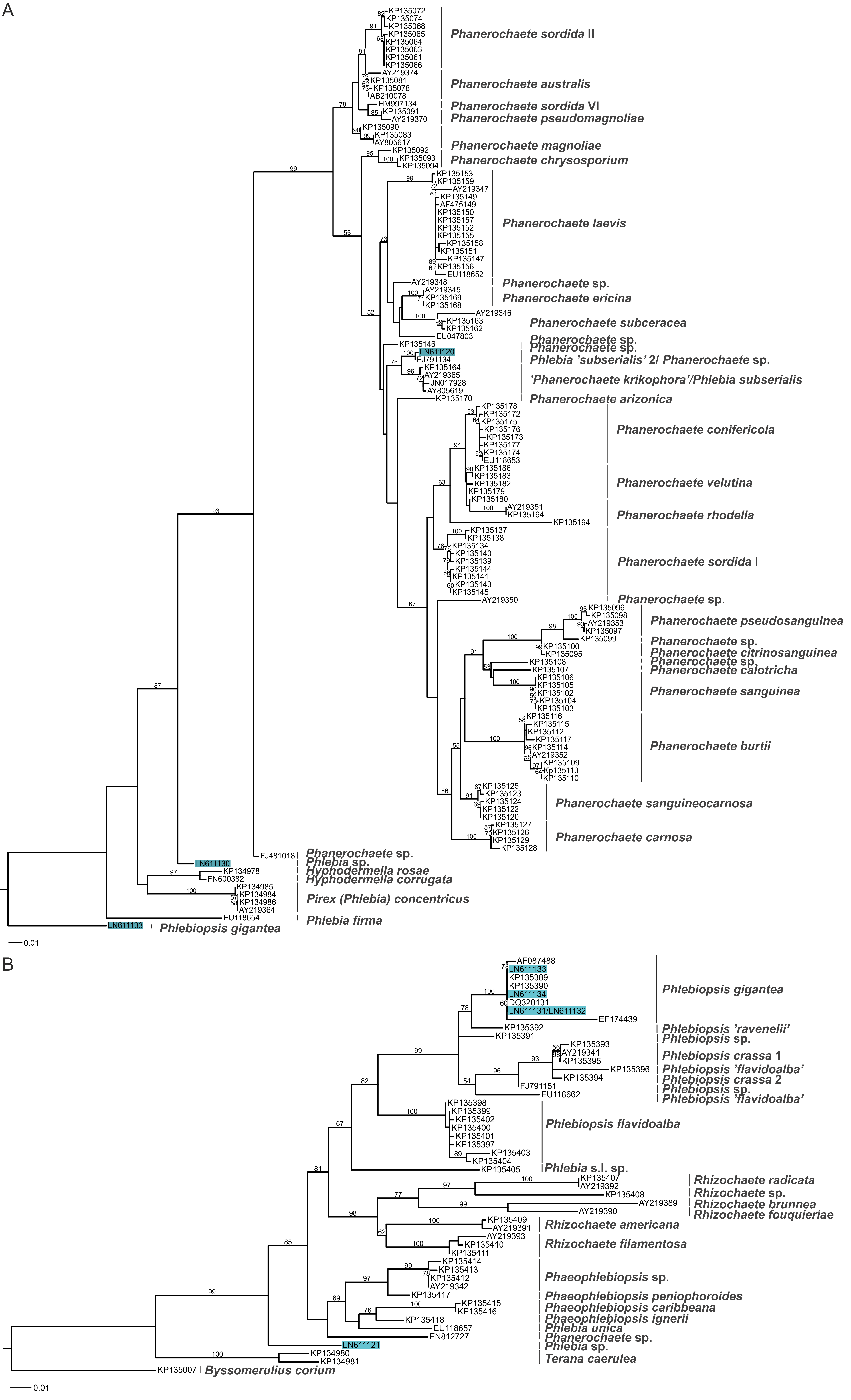

Supplement: Additional file 2: Figure S1. — Maximum likelihood analysis of ITS sequences of (a) Phanerochaete and (b) Phlebiopsis lineages of the phlebioid clade. Description: Bootstrap values (100 replications) higher than 50 % are indicated for the nodes. Fungi of this study (shaded in blue) are compared with related taxons with sequences retrieved from NCBI (http://www.ncbi.nlm.nih.gov/) database. Quotation marks represent uncertain identification or provisional names [20]. Scale bar represents 0.01 nucleotide substitutions per position. (TIFF 4093 kb) [file 12866_2015_538_MOESM2_ESM.tiff]

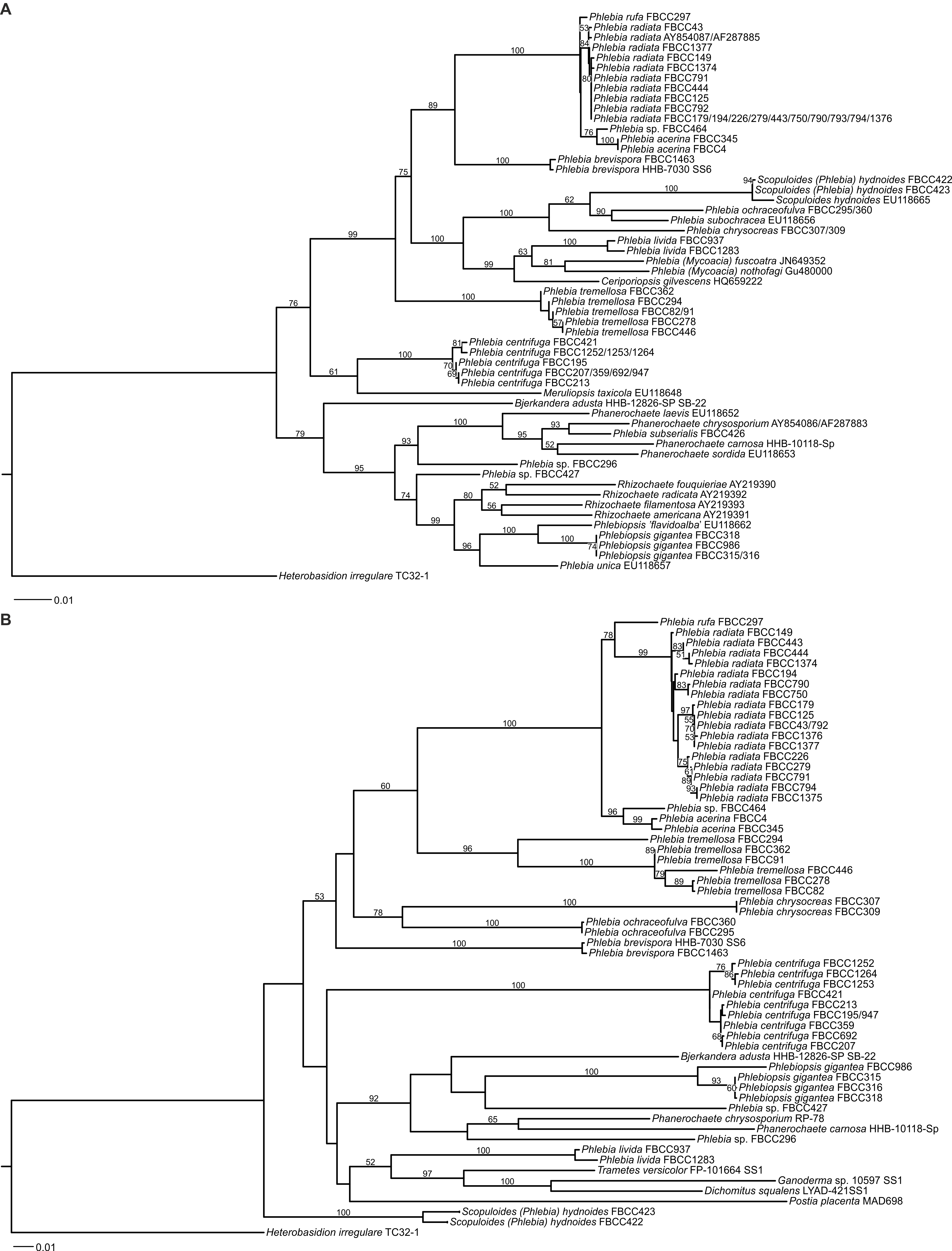

Supplement: Additional file 3: Figure S2. — Maximum likelihood tree of Phlebia isolates and related species. Description: Partial nucleotide sequences from (a) rRNA-encoding genes (ITS1-5.8S-ITS2, LSU) and (b) two protein-encoding genes (gapdh, rpb2) were concatenated for alignment, and the phylogenetic analysis was performed using RAxML v. 7.2.8. and 100x bootstrapping for the nodes. For comparison, sequences from JGI MycoCosm database [76] and NCBI were retrieved. Species names are followed by isolate culture collection identifiers or sequence accessions. Bootstrap values higher than 50 % are indicated for the nodes. Scale bar represents 0.01 nucleotide substitutions per position. (TIFF 3310 kb) [file 12866_2015_538_MOESM3_ESM.tiff]

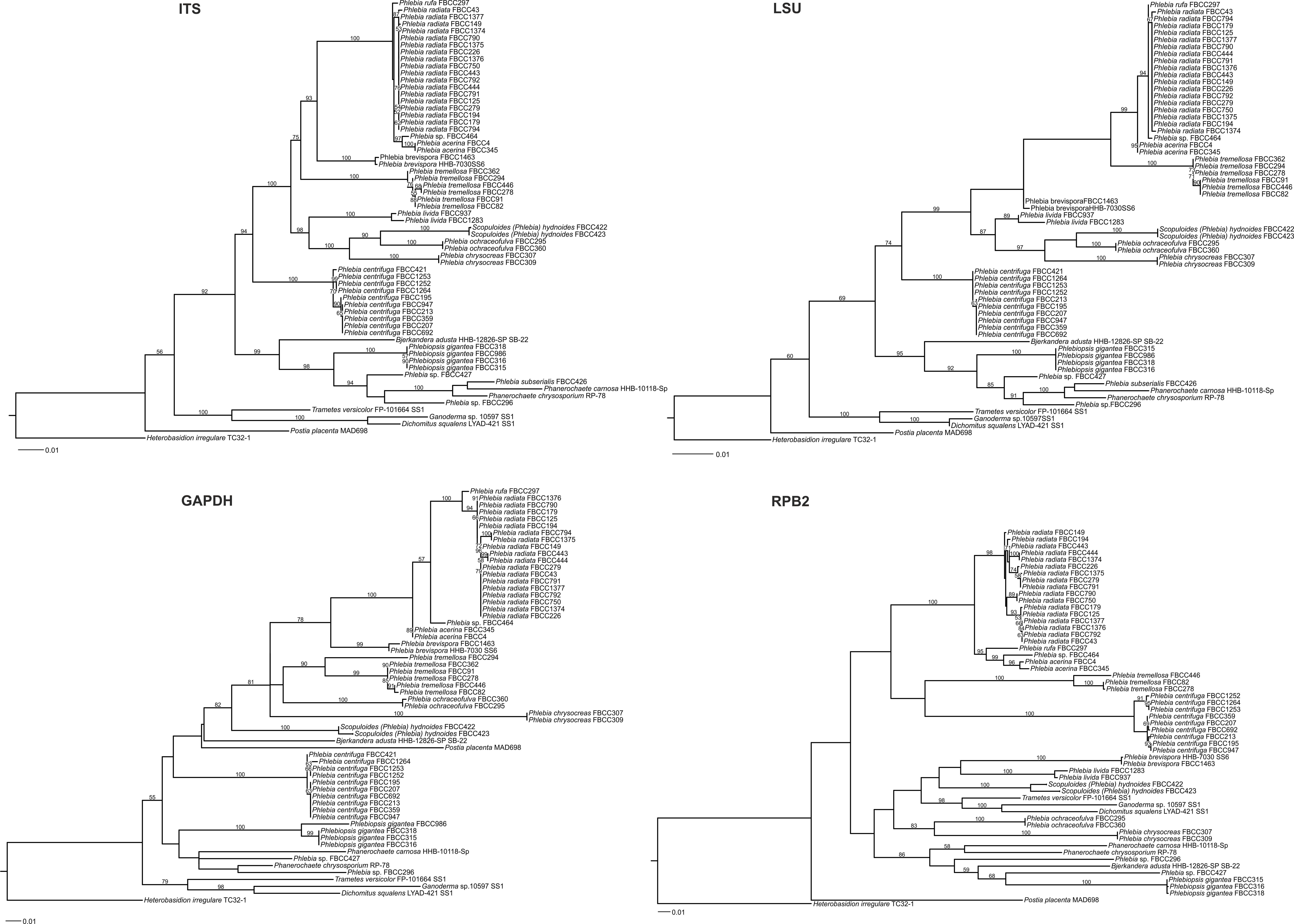

Supplement: Additional file 4: Figure S3. — Phylogenetic trees of phlebioid isolates from maximum likelihood analyses of individual gene datasets. Description: Bootstrap values (100 replications) higher than 50 % are indicated for the nodes. Species names are followed by culture collection identifiers. For comparison, sequences from JGI MycoCosm database [76] were retrieved. Scale bar represents 0.01 nucleotide substitutions per position. (TIFF 6079 kb) [file 12866_2015_538_MOESM4_ESM.tiff]

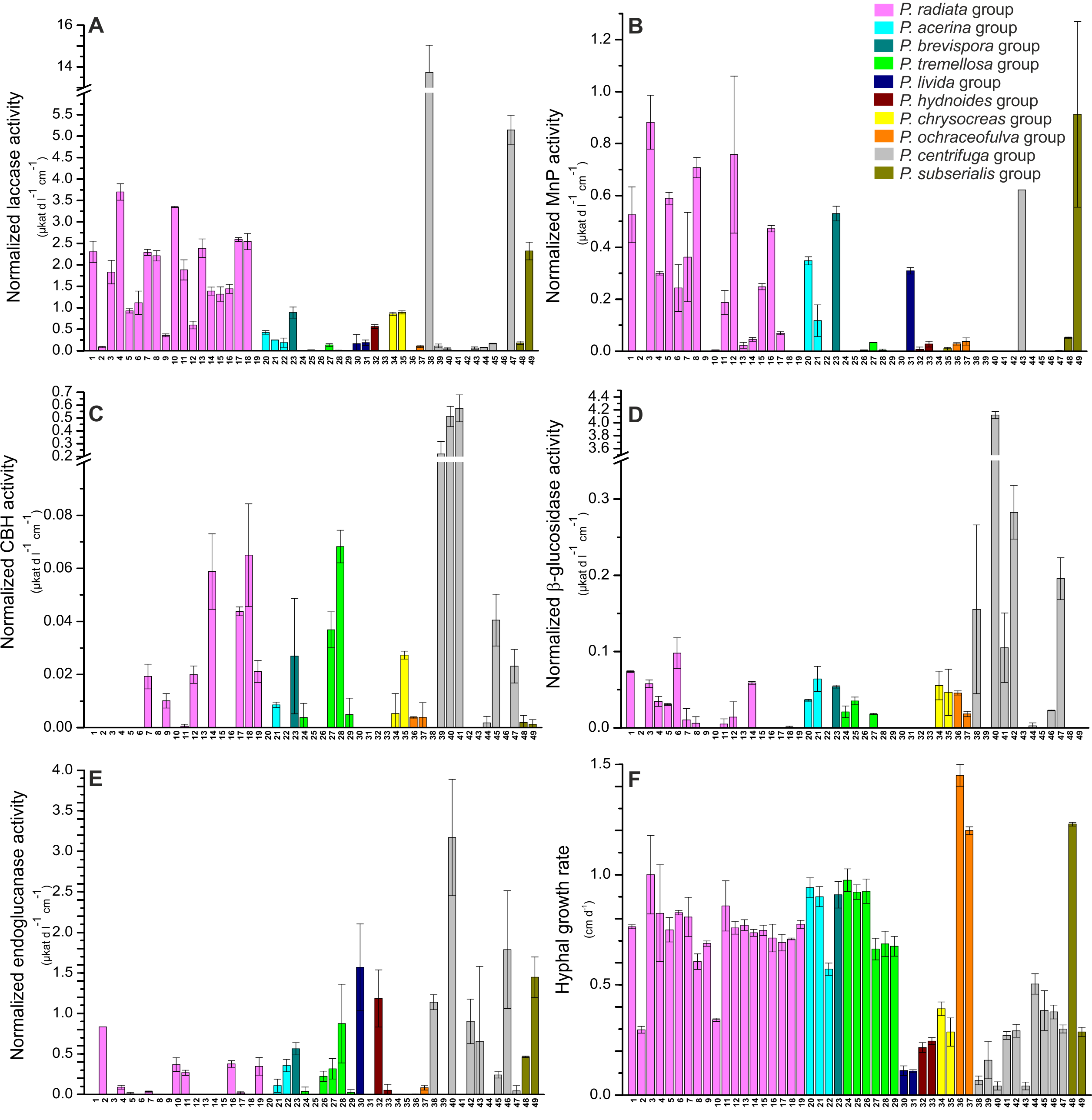

Supplement: Additional file 5: Figure S4. — Normalized enzyme activities of culture liquids and hyphal growth rates of Phlebia isolates. Description: Normalized extracellular (a) laccase (b) MnP (c) CBH (d) β-glucosidase and (e) endoglucanase activities on day 14 in semi-solid milled spruce cultures of Phlebia isolates. Error bars represent standard deviation of the mean activity value from two parallel cultivations. (f) Hyphal growth rates from three parallel MEA plates. Mean value for each isolate is presented. Error bars represent variance of the growth rates. The isolates were numbered as listed in Table 1. (TIFF 1427 kb) [file 12866_2015_538_MOESM5_ESM.tiff]
