# Supplementary material for: Lignocellulose-converting enzyme activity profiles correlate with molecular systematics and phylogeny grouping in the incoherent genus Phlebia (Polyporales, Basidiomycota)
Source: BMC Microbiol. 2015 Oct 19;15:217. doi: 10.1186/s12866-015-0538-x (PMC4610053; doi:10.1186/s12866-015-0538-x)
Supplement: Additional file 6: Table S2. — Statistical tests of model effects and estimates of index parameter in Tweedie distribution. (PDF 155 kb) [file 12866_2015_538_MOESM6_ESM.pdf]

Table S2. Statistical tests of model effects and estimates of index parameter in Tweedie distribution.

Laccase		
	Wald Chi-Square	Sig.
Time	59.483	0.000
Group	102.478	0.000
Time*Group	3656.442	0.000
MnP		
Time	Wald Chi-Square	Sig.
Group	38.287	0.000
Time*Group	331.070	0.000
	9146.242	0.000
CBH		
	Wald Chi-Square	Sig.
Time	59.483	0.000
Group	102.478	0.000
Time*Group	3656.442	0.000
β-glucosidase		
	Wald Chi-Square	Sig.
Time	59.483	0.000
Group	102.478	0.000
Time*Group	3656.442	0.000
Endoglucanase		
	Wald Chi-Square	Sig.
Time	59.483	0.000
Group	102.478	0.000
Time*Group	3656.442	0.000

Enzyme	Tweedie
Laccase	1.677475
MnP	1.625354
CBH	1.445152
β-glucosidase	1.438485
Endoglucanase	1.482727
