# Supplementary material for: Lignocellulose-converting enzyme activity profiles correlate with molecular systematics and phylogeny grouping in the incoherent genus Phlebia (Polyporales, Basidiomycota)
Source: BMC Microbiol. 2015 Oct 19;15:217. doi: 10.1186/s12866-015-0538-x (PMC4610053; doi:10.1186/s12866-015-0538-x)
Supplement: Additional file 7: Table S3. — Accessions for nucleotide and protein-encoding gene model sequences used for comparison in the four-gene phylogenetic analyses. Description: All the sequences were retrieved from JGI MycoCosm database [76] with minor exception: a from NCBI http://www.ncbi.nlm.nih.gov/. (PDF 13 kb) [file 12866_2015_538_MOESM7_ESM.pdf]

Table S3. Accessions for nucleotide and protein-coding gene model sequences used for comparison in the four-gene phylogenetic analyses. All the sequences were retrieved from JGI MycoCosm database [76] with minor exception: [a] from NCBI http://www.ncbi.nlm.nih.gov/

Fungi	SSU	LSU	*gapdh*	*rpb2*
Bjerkandera adusta HHB-12826-SP SB-2	scaffold_116:11112-11672	scaffold_116:6879-11111	jgi|Bjead1_1|183636|estExt_Genemark1.C_140109	jgi|Bjead1_1|163844|gm1.283_g
Phanerochaete chrysosporium RP-78 / *Phanerochaete chrysosporium* isolate AFTOL-ID 776	AY854086[a]	AF287883[a]	jgi|Phchr1|132198|e_gww2.9.328.1	jgi|Phchr1|7545|fgenesh1_pg.C_scaffold_13000333
Phanerochaete carnosa HHB-10118-Sp	scaffold_28:3285-3898	scaffold_28:1934-3284	jgi|Phaca1|261269|fgenesh2_kg.8_#_864_#_scaffold_160_3189_4734	jgi|Phaca1|247866|fgenesh2_kg.1_#_1876_#_isotig11261
Postia placenta MAD-698-R-SB12	scaffold_59:2784-3397	scaffold_59:3398-4810	jgi|PosplRSB12_1|1185348|estExt_Genewise1Plus.C_16_t10169	jgi|PosplRSB12_1|1175997|estExt_Genewise1Plus.C_1_t30126
Trametes versicolor FP-101664 SS1	scaffold_24:1316-1883	scaffold_24:1-15649	jgi|Trave1|170525|estExt_Genewise1Plus.C_8_t20209	jgi|Trave1|110266|e_gw1.1.3121.1
Ganoderma sp. 10597 SS1	scaffold_21:13398-13988	scaffold_21:12699-13397	jgi|Gansp1|154547|gm1.7246_g	jgi|Gansp1|159732|estExt_Genemark1.C_1_t20266
Dichomitus squalens LYAD-421 SS1	scaffold_168:626-1170 + scaffold_162:4601-5120	scaffold_162:3989-4600/scaffold_154:1265-1408	jgi|Dicsq1|183206|estExt_Genemark1.C_540047	jgi|Dicsq1|164685|gm1.454_g
Phlebia brevispora HHB-7030 SS6	scaffold_97:14645-15148 + scaffold_97:17080-17577	scaffold_97:15729-17079	jgi|Phlbr1|163617|estExt_Genemark1.C_170071	jgi|Phlbr1|159852|estExt_Genemark1.C_4_t10175
Heterobasidion irregulare TC32-1	scaffold_09:1649857-1650425	scaffold_09:1650426-1651779	jgi|Hetan2|419475|fgenesh1_pm.07_#_336	jgi|Hetan2|413386|fgenesh1_pm.01_#_528
